# Effects of Birth Weight and Postnatal Nutritional Restriction on Skeletal Muscle Development, Myofiber Maturation, and Metabolic Status of Early-Weaned Piglets

**DOI:** 10.3390/ani10010156

**Published:** 2020-01-16

**Authors:** Liang Hu, Xie Peng, Fei Han, Fali Wu, Daiwen Chen, De Wu, Takele Feyera, Keying Zhang, Lianqiang Che

**Affiliations:** 1 Institute of Animal Nutrition, Sichuan Agricultural University, No.211 Huimin Road, Wenjiang District, Chengdu 611130, Sichuan, China; 2 Department of Animal Science, Aarhus University, DK-8830 Tjele, Denmark

**Keywords:** light weaning weight, nutrient intake, piglets, skeletal muscle, suckling

## Abstract

**Simple Summary:**

Light weaning weight piglets have slow post-weaning growth performance and require longer days to reach slaughter weight. Birth weight and early postnatal nutrient intake are the main factors contributing to light weaning weight. Our results suggested that intrauterine growth-retarded (IUGR) piglets fed adequately during the suckling period do not catch up with the same muscle growth and development compared with normal birth weight piglets. Postnatal early nutritional restriction resulted in impaired skeletal muscle growth and delayed myofiber maturation of the piglets.

**Abstract:**

Piglets with light weaning weight commonly have a slow post-weaning growth rate due to impaired skeletal muscle development. Therefore, the present study aimed to investigate the impact of birth weight and nutrient intake on skeletal muscle development, myofiber maturation, and metabolic status of early-weaned piglets. Twelve pairs of normal birth weight and intrauterine growth-retarded (IUGR) piglets (seven days old) were randomly assigned to receive adequate nutrient intake or restricted nutrient intake for 21 days. Serum and muscle samples were collected for further analysis. The results indicated that muscle weight, cross-sectional areas, and muscular glycogen were lower (*p* < 0.05) in both IUGR and restricted fed piglets. Nutrient restriction decreased the contents of RNA, the RNA to DNA ratio, and the percentages of myosin heavy chain (MyHC) IIx (*p* < 0.05), whereas increased the activity of β-hydroxy-acyl-CoA-dehydrogenase (HAD), the ratio of HAD to citrate synthase, as well as the percentages of MyHC I (*p* < 0.05). In addition, nutrient restriction significantly decreased muscular glycogen, mRNA levels of fatty acid transport protein 1, cationic amino acid transporter 1, and glucose transporter 4 in IUGR piglets compared with the other groups (*p* < 0.05). The results of the present study showed that IUGR impaired skeletal muscle growth and disturbed the hormone and mRNA expression of genes related to energy metabolism, which led to a more severe energy deficit when receiving postnatal nutritional restriction. Postnatal nutritional restriction resulted in delayed myofiber maturation of the piglets, which may be associated with the transformation of MyHC isoform and the change of metabolic status.

## 1. Introduction

Genetic improvement in pig production led to a dramatic increase in litter size over the last decades [1]. Such an increase in litter size results in uterine crowding and limits intrauterine nutrient supply for the growing fetuses that induce intrauterine growth-retarded (IUGR) piglets [2]. Intrauterine growth-retarded piglets are compromised physiologically due to perinatal organ retardation as well as physically due to suckling competition with littermates of normal birth weight (NBW). Moreover, milk intake per individual piglet decreases as litter size increases in modern hyper-prolific sows, which in turn worsens the suckling competition for IUGR piglets. Owing to this fact, several studies reported light weaning weight in IUGR piglets compared with NBW piglets [3,4,5]. Piglets with light weaning weight commonly have a slow post-weaning growth rate and they need longer days to reach slaughter weight relative to piglets with heavier weaning weight due to impaired skeletal muscle development [6]. In our previous study, we have demonstrated the positive impact of nutritional intervention during the early postnatal period on skeletal muscle development and myofiber maturation of the piglets [7]. However, information is lacking whether nutrient restriction could also affect skeletal muscle development and myofiber maturation in piglets with light weaning weight, especially in IUGR piglets.

Skeletal muscles are the key components of growth and play important roles in many physiological activities, such as locomotion, postural maintenance, breathing, and thermogenesis [8,9]. Furthermore, skeletal muscles exhibit remarkable flexibility in the usage of fuel in response to the nutrient intake and energy demands of the organism’s adaptation to a variety of conditions, including disease, stress, and reduced nutrient availability [9,10]. It has been well established that the physiological activity of skeletal muscle is mostly determined by the total fiber number and fiber type composition [11]. Numerous studies have shown that IUGR impairs the growth and development of skeletal muscle, which resulted in a decreased muscle mass as well as delayed skeletal muscle maturity [12,13]. However, most of the studies that investigated the influence of IUGR on muscle myofiber development were focused on exploring post-weaning stages regardless of early nutritional intervention [14,15]. Therefore, it is highly relevant to clarify whether early nutritional intervention, adequate vs. restricted, would have a differential impact on the skeletal muscle development and myofiber maturation in light weaning weight IUGR piglets that received the nutritional intervention during the early postnatal period. Moreover, the metabolic status of skeletal muscles in piglets with IUGR and/or restricted postnatal nutrition is merely unknown. Therefore, the present study aimed to investigate the impact of birth weight and postnatal nutritional restriction on muscle development, myofiber maturation, and metabolic status of early-weaned piglets.

## 2. Materials and Methods

The present experiment complied with the law of animal protection and was approved by the Animal Care and Use committee of the Sichuan Agricultural University (Ethic Approval Code: SCAUAC201408-3) and was performed in accordance with the National Research Council’s Guide for the Care and Use of Laboratory Animals.

### 2.1. Experimental Design, Formula Milk, and Animal Management

Data used in the present study were collected from a previous animal experiment in which the detailed description of the experimental animals and diets were published elsewhere [16]. In brief, piglets with ±0.5 SD birth weight to the mean litter birth weight were categorized as NBW piglets, whereas those with at least 1.5 SD lower than the mean litter birth weight were categorized as IUGR piglets as described previously [17,18]. Moreover, piglets defined as IUGR had a steep, dolphin-like forehead and either bulging eyes and/or hair with no direction of growth according to Hales et al. [19]. Based on the criterions of birth weight and head shape, accordingly, 12 pairs of newborn boars (Pig Improvement Company 327 × 1050) of NBW piglets (1.45–1.59 kg) with a body weight (BW ± SD) of 1.56 ± 0.05 kg and IUGR piglets (0.88–0.93 kg) with BW ± SD of 0.91 ± 0.03 kg, respectively, were selected from 12 healthy sows that had the same litter size, i.e., 10 live born piglets per litter. All piglets used in this experiment were weaned at 7 d of age and moved to individual small metabolism cages (0.8 m × 0.7 m × 0.4 m) where the formula milk was fed to the piglets for 21 days of the experimental period every 3 h by a bottle feeding system between 06:00 and 24:00. The formula milk was prepared by dissolving 1 kg of formula powder (DM 87.5%) in 4 L of water so that the final formula milk should have nearly similar nutrient composition as sow milk (Supplementary Table S1). In this experiment, six pairs of NBW and IUGR piglets, respectively, were randomly assigned to receive adequate nutrient intake (ANI), whereas the other six pairs NBW and IUGR piglets, respectively, were randomly assigned, to receive restricted nutrient intake (RNI). Thus, 2 × 2 factorial experiments were employed in this investigation forming the following combinations: NBW-ANI, NBW-RNI, IUGR-ANI, and IUGR-RNI (*n* = 6, per group). Among them, NBW-ANI and IUGR-ANI piglets had formula milk ad libitum, NBW-RNI piglets were provided the same amount of formula milk as IUGR-ANI piglets, while IUGR-RNI piglets were provided approximately 70% of formula milk intake by IUGR-ANI piglets. Therefore, the ANI groups had ad libitum formula milk while the RNI groups were restricted by 30% of the ad libitum formula milk compared to the ANI groups during the 21 d of experimental period. Piglets had free access to water. The ambient temperature and humidity were controlled around 30 °C and 50–60%, respectively.

### 2.2. Blood and Tissue Samples Collection

Blood samples were collected by precaval vein puncture at the end of the experimental period before the morning meal (08:00) with an overnight fasting. The collected blood samples were allowed to coagulate for 40 min before centrifugation (3500× *g*, 10 min, 4 °C), and serum samples were harvested and kept at −20 °C for pending analysis. Following blood sampling, all piglets were euthanized with an intravenous injection of pentobarbital sodium (50 mg/kg BW) and slaughtered. Then, the body weight and brain weight of all piglets were weighed immediately. The semitendinosus (ST) and psoas major (PM) muscles were completely excised from the left side of the carcass and their weight and length were recorded, respectively. Muscle samples for histological analyses were taken from the central part of the PM muscle and then stored in 4% methanol solution. Moreover, approximately 8 g of the PM muscle samples were collected for gene expression, snap-frozen in liquid nitrogen, and stored at −80 °C for later analysis.

### 2.3. Determination of Serum Hormones

Serum insulin (catalogue no. F01TB) and leptin (catalogue no. KAP2281) concentrations were measured by a commercial multi-species RIA kit (Beijing North Institute of Biotechnology, Beijing, China) validated for measuring porcine serum samples with the detection limit of 1.0 μIU/mL and 0.2 ng/mL for insulin and leptin, respectively. The intra- and inter-assay coefficient of variations for insulin and leptin were 5% and 10%, respectively. Serum total triiodothyronine (T3; catalogue no. H222) and thyroxine (T4; catalogue no. H223) concentrations were determined by radioimmunoassay using commercially available standard RIA kits (Nanjing Jiancheng Institute of Bioengineering, Nanjing, China) with the minimum detection levels of 0.1 and 0.2 ng/mL for T3 and T4, respectively. Each serum sample was analyzed in duplicate.

### 2.4. Determination of Serum Metabolites

Serum glucose (catalogue no. CH0101105), total cholesterol (TC; catalogue no. CH0101160), triglyceride (TG; catalogue no. CH0101159), high-density lipoprotein-cholesterol (HDL-C; catalogue no. CH0101161), and low-density lipoprotein-cholesterol (LDL-C; catalogue no. CH0101162) were determined by an enzymatic chromatometric method using the corresponding commercial kits (Sichuan Maker Biotechnology Co., Ltd., Sichuan, China) according to the manufacturer’s instructions. The concentration of non-esterified fatty acid (NEFA) was determined by using a commercial enzymatic procedure (NEFA-C Kit catalogue no. 434-91795, ACS-ACOD Method; Wako Chemicals, Neuss, Germany). The intra- and inter-assay coefficient of variation was less than 5% in each of the assay. Serum total amino acids (tAA) were analyzed by the Biochemical Analytical Instrument (Hitachi 7170, Hitachi Limited, Tokyo, Japan). All samples were analyzed in duplicate.

### 2.5. Determination of Psoas Major Muscle Metabolites

Before subsampling the muscle for metabolites analysis, the PM muscle tissues were thoroughly homogenized. The concentrations of TG (catalogue no. A110-1-1), adenosine triphosphate (ATP; catalogue no. A095-1-1), malondialdehyde (MDA; catalogue no. A003-1-2), glycogen (catalogue no. A043-1-1), and lactate (catalogue no. A019-2-1) in the PM muscle were determined using commercial assay kits (Nanjing Jiancheng Institute of Bioengineering, Nanjing, Jiangsu, China) according to the manufacturer’s instructions for each of the metabolites. Briefly, MDA was quantified using the thiobarbituric acid method according to Hu et al. [20]. The concentrations of TG and ATP were determined according to the manufacturer’s instructions on a UV-vis spectrophotometer (UV-1100, Shanghai Mapada Instruments Co., Ltd., Shanghai, China). The concentrations of glycogen and lactate were determined as described previously by Wang et al. [21], and the results were presented as milligram of glycogen or lactate per gram of muscle sample (wet weight). All samples were analyzed in duplicate.

### 2.6. Muscular Morphology

For muscular morphology determination, the PM muscle samples stored in 4% methanol solution were prepared after staining with hematoxylin and eosin using standard paraffin-embedding procedures according to a previous study [7]. All muscular sections were photographed using a digital microscope (Nikon), and the muscle fibers were counted over 5 randomly selected fields of known size (1.01 mm^2^, 200 to 300 fibers) as the myofiber density [22]. For total cross-sectional area (CSA) of the PM muscle, a whole mid-belly slice of approximately 5 mm was rapidly frozen in liquid nitrogen, the muscle CSA was calculated from the circumference of the muscle mid-belly. Then, the estimated total myofiber number was obtained by multiplying the fiber number per unit area with the CSA of the PM muscle. The Myofiber density was used to estimate the total number of fibers by multiplying with the CSA of the PM muscle. The mean muscle fiber diameter in the united area was measured by the Image-Pro Plus 6.0 software (Media Cybernetics, Bethesda, Rockville, MD, USA). The person who was blinded to the experimental protocols and animal experiment conducted microscopic analyses consistently.

### 2.7. Biochemical and Enzyme Analyses

Total RNA of the muscle samples (approximately 100 mg) was extracted using TRIzol reagent (Invitrogen, Carlsbad, CA, USA) according to the manufacturer’s instructions. The quality and purity of the RNA samples were assessed by agarose gel (1.0%) electrophoresis and nucleic acid analyzer (Beckman DU-800; Beckman Coulter, Inc., Brea, CA, USA), respectively. Deoxyribonucleic acid of the muscle samples was extracted using the QIAamp^®^ DNA mini kit (Qiagen) according to the manufacturer’s instructions, and DNA quantification was performed by nucleic acid analyzer. Protein concentration, lactate dehydrogenase (LDH; Catalogue no. A020-2-2), citrate synthase (CS; Catalogue no. A108-2-1), and β-hydroxy-acyl-CoA-dehydrogenase (HAD; Catalogue no. H196) activities of the muscle samples were determined by using commercial kits (Nanjing Jiancheng Bioengineering Institute, Nanjing, China) according to the manufacturer’s instruction manuals. For biochemical and enzymes analyses of the muscle samples, the frozen muscle samples (approximately 50 mg) were homogenized in 450 μL of 0.9% saline solution and then centrifuged at 3500× *g* for 10 min at 4 °C. The protein content in the muscle’s supernatant was determined based on the method of Coomassie brilliant blue dyeing using bovine serum albumin as the standard. The rate of change of absorbance was monitored at 440, 340, 412, and 660 nm for determination of LDH, CS, HAD activities and protein concentration, respectively, using a biochemical analyzer (Multiskan Spectrum, Thermo scientific). The contents of RNA, DNA, and protein were presented as mg/g fresh muscle. The activities of LDH, CS, and HAD were presented as units per gram of protein (U/g protein).

### 2.8. Real-Time Reverse Transcription-PCR (RT-PCR) Analysis

Reverse transcription was performed at 37 °C for 15 min, followed by RT inactivation at 85 °C for 5 s using the PrimeScript^TM^ RT reagent Kit (Catalogue no. RR047A; Takara) according to the manufacturer’s instructions. A portion of the RT products (1 μL) was used directly for RT-PCR analysis. Real-time PCR assays were performed on complementary DNA samples in 384-well optical plates on a 7900HT ABI Prism Sequence Detection System (Applied Biosystems, Foster City, CA, USA) using the SYBR green system (Catalogue no. RR820A; Takara). Primers for individual gene were designed using Primer Express 3.0 (Applied Biosystems) and given in Table 1. The 18 s RNA was identified by using GeNorm as the most stable reference gene in this experiment. The reaction mixture (10 μL) contained 5 μL of freshly SYBR^®^ Premix Ex TaqII (Tli RNaseH Plus) and 0.2 μL ROX Reference Dye II (50×), 0.8 μL of the primers, 1 μL of RT products, and 3 μL diethylpyrocarbonate-treated water. The PCR protocol was used as follows: 1 cycle (95 °C for 30 s), 40 cycles (95 °C for 5 s, 60 °C for 31 s), and 1 cycle (95 °C for 15 s, 60 °C for 1 min and 95 °C for 15 s). The standard curve for each gene was run in duplicate and three times for obtaining reliable amplification efficiency values as described previously [7]. The correlation coefficients of all the standard curves were more than 0.99, the amplification efficiency values were between 90% and 110%. At the end of amplification, dissociation analyses of the PCR product were performed to confirm the specificity of PCR products. The relative mRNA abundance of analyzed genes was calculated using the method of 2^−ΔΔ*C*t^, as described previously [23]. In addition, the percentages of myosin heavy chain (MyHC) isoforms were calculated as the ratio of the normalized expression level of each MyHC isoform to the total expression of MyHC [24].

### 2.9. Statistical Analyses

Data were tested for normality (Shapiro–Wilk test) and homogeneity of the variances (Levene’s test) before statistical analyses. Data of NEFA was transformed before statistical analysis. Data were analyzed using the mixed linear procedure of Statistical Product and Service Solutions (Ver.20.0 for Windows, SPSS, Chicago, IL, USA) according to the following mode: *y_ijk_* = *μ* + *a_i_* + *b_j_* + (*ab*)*_ij_* + *e_ijk_* (*_i_* = 1, 2, *_j_* = 1, 2, *_k_* = 1, 2,…, *n_ij_*), where *y_ijk_* represents the dependent variable, *μ* is the mean, *a_i_* is the effect of BW (IUGR, NBW), *b_j_* is the effect of NI (ANI, RNI), (*ab*)_ij_ is the interaction effect between BW and NI, and *e_ijk_* is the error term. Differences were considered significant when *p* < 0.05, and a tendency was recognized when 0.05 < *p* ≤ 0.10. When the statistical test was significant, means were compared using Tukey’s multiple comparisons test. Results were presented as means ± SEM. Interactions between BW and NI were not significant for most of the parameters considered. Thus, only the main effects were presented in tables, while means for significant interactions were presented as figures.

## 3. Results

### 3.1. Muscle Weight

Compared with NBW piglets, IUGR piglets had lower weight and length of PM and ST, respectively, as well as a lower ratio of PM and ST to BW (*p* < 0.05). In contrast, IUGR piglets had greater brain to PM and ST weight ratio (*p* < 0.05; Table 2). Restricted formula milk intake decreased the weight and length of PM and ST, as well as the relative weight of PM and ST to BW (*p* < 0.05), but increased the ratio of brain to PM (*p* < 0.05; Table 2).

### 3.2. Serum Biochemical Properties

**Figure 1:**
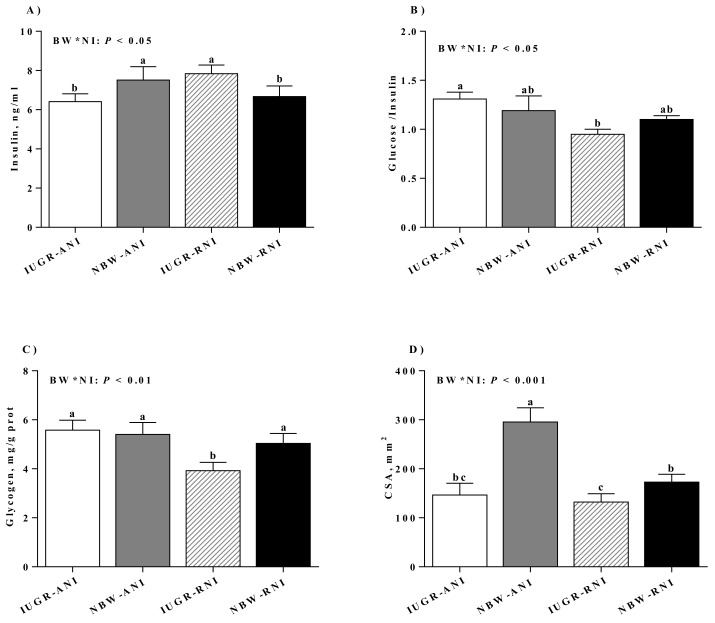

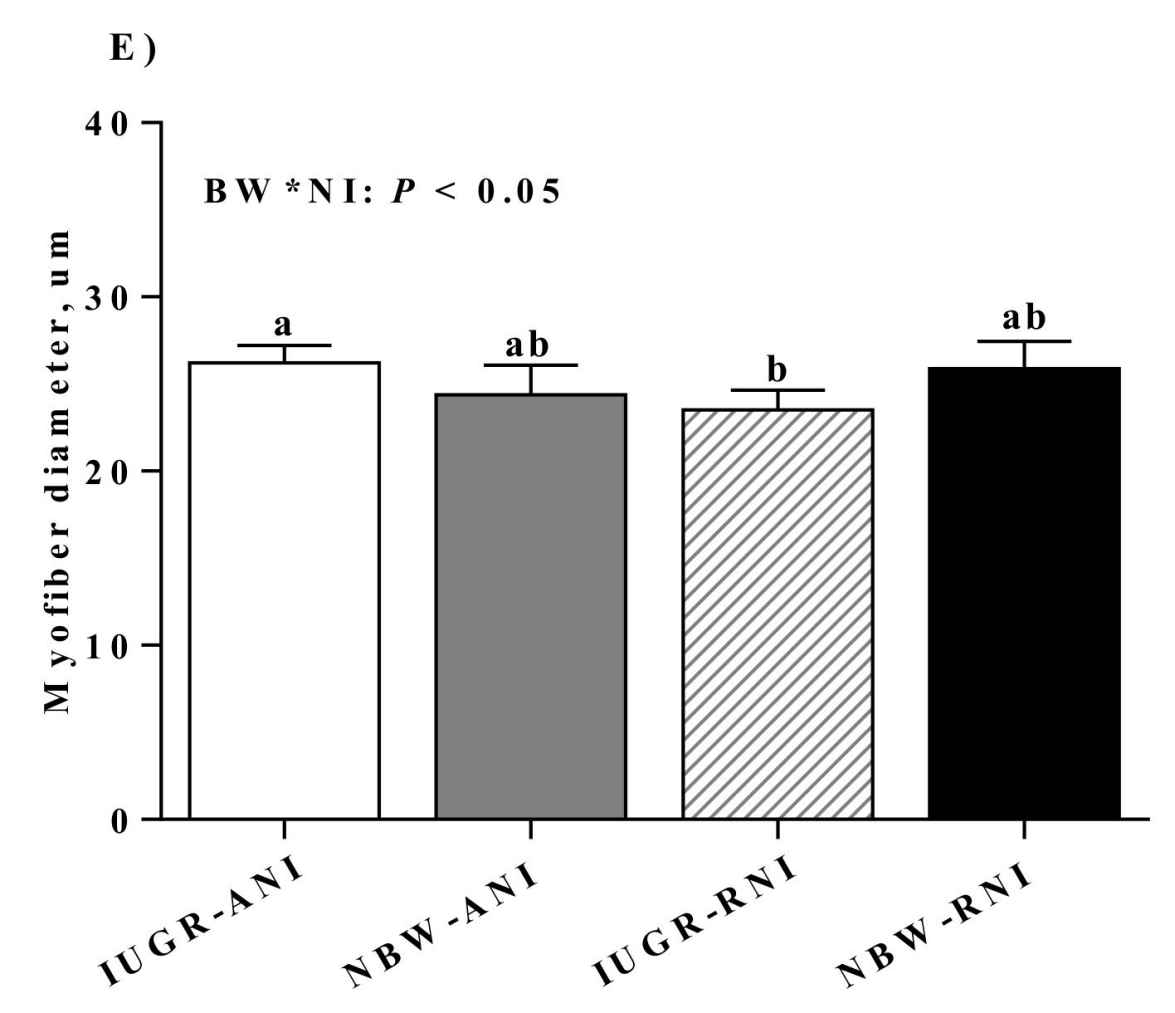
The interactive effects of nutrient intake on (**A**) serum insulin, (**B**) the ratio of glucose to insulin, (**C**) psoas major muscular glycogen, (**D**) CSA, (**E**) myofiber diameter of intrauterine growth-retarded (IUGR), and normal-birth weight (NBW) piglets. ANI, adequate nutrient intake; RNI, restricted nutrient intake; BW, birth weight; NI, nutrient intake; CSA, cross-sectional area. ^a,b,c^ Means within a trait with different superscripts differ (*p* < 0.05).

Intrauterine growth-retarded piglets had greater tAA (*p* < 0.05) but lower TC (*p* = 0.05) contents in the serum than the NBW piglets (Table 3). Irrespective of BW, RNI decreased the concentrations of TG and TC (*p* < 0.05) as well as the ratio of T3 to T4 (*p* = 0.03) in piglets’ serum (Table 3). Moreover, RNI tended to decrease serum concentrations of glucose (*p* = 0.06) and T3 (*p* = 0.06). Interactions (*p* < 0.05) between BW and NI were observed for insulin concentration (*p* < 0.05; Figure 1A) and the ratio of glucose to insulin (*p* < 0.05; Figure 1B) in the piglets’ serum.

### 3.3. Muscular Metabolites

Intrauterine growth-retarded piglets had lower muscle glycogen and ATP contents than the NBW piglets (*p* < 0.05; Table 4). Regardless of BW, RNI decreased the concentration of muscle glycogen (*p* < 0.05) and tended to decrease the concentration of muscle TG (*p* = 0.06; Table 4). Interaction between BW and NI was observed for muscle glycogen concentration (*p* < 0.05) in the PM muscle of the piglets (Figure 1C).

### 3.4. Muscle Characteristics

Regardless of the NI, IUGR piglets had smaller CSA (*p* < 0.001) and myofiber number (*p* = 0.003) of PM muscles than the NBW piglets (Table 5). Irrespective of the BW, RNI decreased CSA of PM muscles in the piglets (*p* < 0.05) and tended to decrease myofiber number (*p* = 0.08), but tended to increase myofiber density (*p* < 0.05; Table 5). Interactions between BW and NI were observed for CSA (*p* < 0.001; Figure 1D) and myofiber diameter (*p* = 0.03; Figure 1E) of the PM muscles.

### 3.5. The Contents of RNA, DNA, and Protein in Muscles

Regardless of the NI, BW had no influence (*p* > 0.05) on the contents of RNA, DNA, and protein in the muscle (Table 6). However, RNI decreased the contents of RNA and the ratio of RNA to DNA of the PM muscle (*p* < 0.05), and tended to decrease the content of DNA in the PM muscle (*p* = 0.10; Table 6). Tendencies of interactions between BW and NI were observed for RNA (*p* = 0.10; Table 6) and the ratio of RNA to DNA (*p* = 0.06; Figure 1F).

### 3.6. Metabolic Enzyme Activities

Regardless of BW, RNI increased the activity of HAD (*p* = 0.003) and the ratio of HAD to CS (*p* < 0.05), but decreased the ratio of LDH to HAD (*p* = 0.04; Table 7) in the PM muscle.

### 3.7. mRNA Expressions and Percentages of Myosin Heavy Chain Isoform

Regardless of BW, RNI increased mRNA expression of the MyHC I in the PM muscle of piglets (*p* = 0.009; Table 8). Moreover, RNI increased the percentage of MyHC I (*p* = 0.04) but decreased the percentages of MyHC IIx (*p* = 0.03) in the PM muscle (Table 8). Restricted formula milk intake tended to decrease the percentage of MyHC IIb in the PM muscle (*p* = 0.07; Table 8).

### 3.8. mRNA Expressions of Genes Related with Amino Acid, Glucose, and Fatty Acid

**Figure 2:**
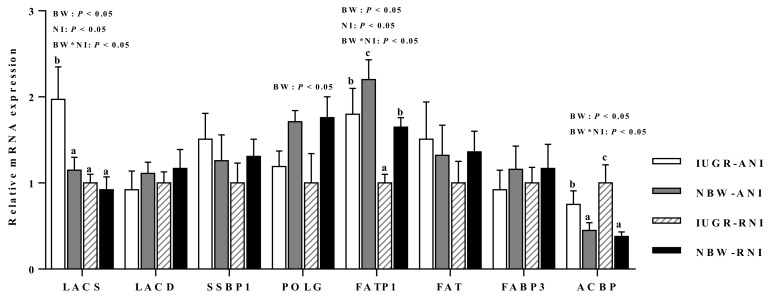
Effects of the level of nutrient intake on lipids metabolism related genes expression in psoas major muscles of intrauterine growth-retarded (IUGR) and normal-birth weight (NBW) piglets. ANI, adequate nutrient intake; RNI, restricted nutrient intake; BW, birth weight; NI, nutrient intake; *LACS*, long-chain acyl-CoA synthetase; *LACD*, long-chain acyl-CoA dehydrogenase; *SSBP1*, single-stranded DNA-binding protein 1; *POLG*, DNA polymerase gamma; *FATP1*, fatty acid transport protein 1; *FAT*, fatty acid translocase; *FABP3*, fatty acid binding protein 3; *ACBP*, acyl-CoA binding protein. ^a,b,c^ Means within a gene with different superscripts differ (*p* < 0.05). Effect of birth weight was significant for *LACS*, *POLG*, *FATP1*, and *ACBP* (*p* < 0.05). Effect of NI was significant for *FATP1* and *LACS* (*p* < 0.05). Interactions between birth weight and NI were significant for mRNA abundance of *FATP1*, *ACBP*, and *LACS* (*p* < 0.05).

**Figure 3:**
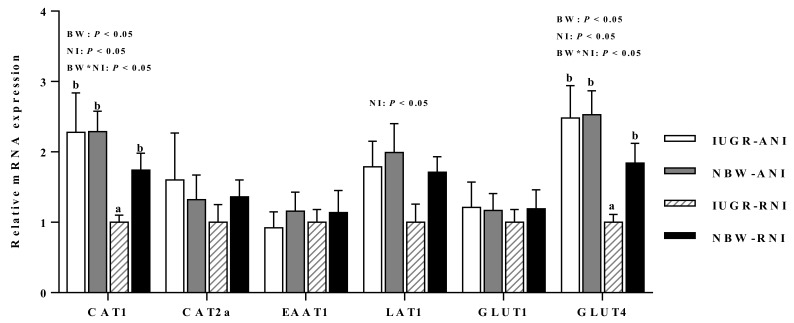
Effects of the level of nutrient intake on amino acids and glucose metabolism related genes expression in PM muscles of intrauterine growth-retarded (IUGR) and normal-birth weight (NBW) piglets. ANI, adequate nutrient intake; RNI, restricted nutrient intake; BW, birth weight; NI, nutrient intake; PM, psoas major muscle; *GLUT*, glucose transporter; *CAT*, cationic amino acid transporter; *EAAT1*, excitatory amino acid transporter1; *LAT1*, L-type amino acid transporter 1. ^a,b^ Means within a gene with different superscripts differ (*p* < 0.05). Effect of birth weight was significant for *CAT1* and *GLUT4* (*p* < 0.05). Effect of NI was significant for *CAT1*, *GLUT4*, and *LAT1* (*p* < 0.05). Interactions between birth weight and NI were significant for mRNA abundance of *CAT1* and *GLUT4* (*p* < 0.05).

Intrauterine growth-retarded piglets had greater mRNA abundance of long-chain acyl-CoA synthetase (LACS; *p* < 0.05) and acyl-CoA binding protein (ACBP; *p* < 0.05), but had lower abundance of DNA polymerase gamma (POLG; *p* < 0.05), fatty acid transport protein 1 (FATP1; *p* < 0.05), cationic amino acid transporter 1 (CAT1; *p* < 0.05), and glucose transporter 4 (GLUT4; *p* < 0.05) than the NBW piglets (Figure 2 and Figure 3). Postnatal nutrient restriction decreased mRNA abundance of LACS, FATP1, cationic amino acid transporter 1 (CAT1; *p* < 0.05), glucose transporter 4 (GLUT4; *p* < 0.05), and L-type amino acid transporter 1 (LAT1; *p* < 0.05) in the PM muscle of IUGR piglets than the NBW piglets (Figure 2 and Figure 3). In addition, interactions between BW and NI were observed for LACS, FATP1, ACBP, CAT1, and GLUT4 (*p* < 0.05).

## 4. Discussion

High body weight at weaning is an important determinant of growing/finishing performance in the pig industry as weaning weight directly influences post-weaning growth performance. Skeletal muscle development is the key component of growth and development in pigs. The present study was designed to investigate the impact of early postnatal nutritional intervention on skeletal muscle development and myofiber maturation in IUGR piglets to delineate the role of birth weight from postnatal nutritional intervention.

Several studies reported that IUGR negatively affects the growth and development of skeletal muscle in pigs [13,25]. Furthermore, a defect in normal muscle development during the neonatal period can influence later growth and health [26]. In this study, both IUGR and RNI significantly decreased the weight and length of PM and ST muscles, which supported the compromised growth performance we reported previously [16]. Interactions between BW and NI were rarely prevalent on most of the dependent variables investigated in the present study. The lack of such interactions implied that feeding IUGR piglets adequately does not enable them to catch up with similar muscle growth and development as that of NBW piglets. Thus, the results of the present study evidenced the dominance of birth weight over nutritional planning in regulating postnatal muscle growth and development in IUGR piglets. Skeletal muscle development is less prioritized than other key organs such as the brain and heart in a situation when nutrients are not adequately provided [27]. The present study also supported this finding in that IUGR piglets had lower absolute muscle weight but greater brain to muscle weight than NBW piglets, indicating that brain development was highly prioritized in IURG piglets. Numerous studies have shown that IUGR piglets have a smaller muscle mass than the contemporary NBW piglets [25,28], reflecting a decreased muscle growth prenatally and/or postnatally [29,30].

The total myofiber number and CSA of the muscle are known as the key determinants of postnatal muscle growth [7,31]. Consistent with these findings, the results of the present study demonstrate lower CSA and myofiber number of PM induced by IUGR and/or nutrient restriction coincided with muscle weight of piglets, which indicated that pre- and/or post-natal malnutrition had negative impacts on total myofiber number and CSA [5,26]. Moreover, the content of RNA and ratio of RNA:DNA of the PM muscle, which is a potential index of protein synthesis activity [32], were lowered by nutrient restriction, reflecting a decreased protein synthesis rate of the piglets.

It has been well known that restricted muscle development in the early postnatal period could permanently alter growth performance and metabolic maturation during the later stage of life [33,34]. Except for the greater concentration of HAD induced by nutrient restriction, other enzymatic activities in the muscle did not differ between NBW and IUGR piglets. The increased activity of HAD and the ratio of HAD to CS observed in the present study indicates increased lipid β-oxidation capacity of the PM muscle in piglets received restricted nutrient postnatally. However, the activity of LDH was neither affected by birth weight nor nutrient intake in the present study, suggesting that the glycolytic capacity was preserved, which is inconsistent with a previous study [8]. This discrepancy could be attributable to the stage of the experiment or the severity of the undernutrition. The lower serum glucose concentration detected in the present study in RNI piglets reflects the shift in the oxidation pattern of the piglets from glucose to fatty acids, which was induced by nutrient restriction during the early postnatal period. The types of muscle fibers largely depend on the types of MyHC existing in the muscle [35]. Rapid myofibril protein accretion and changes in MyHC polymorphism of myofibers in the skeletal muscle of piglets have been reported to occur during the neonatal period [11]. Restricted nutrient intake resulted in a greater percentage of MyHC I and a lower percentage of MyHC IIx type muscle fiber of the PM muscle in both IUGR and NBW piglets at mRNA level, which need further investigation. In support of the present results, previous studies have shown that both prenatal and postnatal nutritional restriction could potentially induce an increased oxidative capability in favor of slow-oxidative fibers at the expense of fast-glycolytic fibers [36,37]. In addition, the reduced T3 content and T3 to T4 ratio induced by RNI were in accordance with the transformation of MyHC isoform. The reduced levels of thyroid hormone cause fast-to-slow shifts in MyHC isoform expression [38].

One of the negative physiological impacts of IUGR piglets is the disturbance of the umbilical vein serum metabolites, which are associated with the disorders of nutrient and energy metabolism as well as endocrine imbalances during the neonatal period [39]. In the current study, IUGR piglets had lower serum concentration of TC and tended to have lower serum concentrations of HDL-C and LDL-C. On the other hand, RNI significantly lowered serum concentrations of TG and TC, which could be associated with the lower expressions of FATP1 in PM muscles, indicating the lower fatty acid uptake in PM muscles induced by RNI. Notably, TG can be stored as lipid droplets within hepatocytes or secreted into the blood, but it could also be hydrolyzed and the fatty acids are channeled towards the β-oxidation pathway during undernutrition [40]. Similar metabolic changes were observed in children with marasmus due to postnatal nutritional restriction [41]. Neonates with IUGR tend to develop hyperglycemia and insulin resistance due to impairment of β-cell development [42]. However, we found no difference in glucose homeostasis between NBW and IUGR piglets in the present study. It could be speculated that 28 days are too short for the piglets to detect changes in circulating glucose levels. Interestingly, we found RNI stabilized serum insulin concentration of IUGR piglets at a similar level to NBW piglets with ANI, which was also accompanied by a similar ratio of glucose to insulin. A previous investigation indicated that similar nutritional restriction during the pre- and post-natal period for IUGR individuals leads to normal insulin secretion [43], which is also reflected in the present study between NBW-ANI and IUGR-RNI piglets. Leptin is known to play an important role in the regulation of feed intake and energy partitioning, which is often considered as a marker for adiposity in adult pigs [44]. In the present study, leptin concentration was markedly higher in IUGR than in NBW piglets, which is consistent with previous studies [45,46]. Furthermore, IUGR increased mRNA expression of LACS and ACBP in the PM muscles, indicating that IUGR piglets might use fat as an energy source to a certain extent.

A previous study showed that light weaning weight piglets had a greater protein degradation rate and lower protein synthesis as well as reduced glucose metabolism compared with normal littermates [9]. The present result showed that IUGR piglets had greater serum tAA concentration than NBW piglets. Moreover, RNI was observed to down-regulate gene expression of CAT1a and LAT1 in PM muscles of IUGR piglets. The CAT1a and LAT1 are the carrier proteins mediating the transport of cationic and neutral amino acids, respectively [47]. The increased serum tAA concentration in IUGR piglets and the decreased mRNA expression of AA transporters indicated an increased body protein catabolism or a reduced protein synthesis rate of piglets when suffering from undernutrition. Similarly, Yang et al. has reported that IUGR led to an increase in some of the neutral amino acids contents in plasma, liver, and skeletal muscle compared with their normal littermates, which was accompanied by the inhibition of the mRNA expression of AA transporters [48]. In the present study, mRNA expression of GLUT4 in IUGR-RNI piglets was markedly lower than piglets in other groups. The GLUT4 is a rate-limiting step in glucose uptake and acts as the port of entry for glucose into the muscle [49]. Thus, the lower serum glucose level in RNI piglets and the reduced muscle glycogen concentration in IUGR-RNI piglets denote a marked energy deficit in nutrient restricted IUGR piglets.

Maternal malnutrition induced IUGR has been reported to decrease ATP production through impaired oxidative phosphorylation process and enhanced oxidative stress in the liver and skeletal muscle of the offspring [50]. In agreement with this finding, the present study demonstrated a lower concentration of ATP in the PM muscle of IUGR piglets, which might reflect the impaired TCA cycle, electron transport, and ATP formation [51]. The SSBP1 and POLG, which are responsible for mitochondrial DNA replication and repair, are involved in the TCA cycle and the ATP formation [52]. In addition to the lower ATP content, the expression level of POLG was lower in IUGR piglets regardless of the imposed nutritional levels, emphasizing a reduced oxidative energy metabolism in the PM muscle of IUGR piglets.

## 5. Conclusions

It can be concluded from the present study that IUGR impaired skeletal muscle growth, and disturbed the hormone and mRNA expression of genes related to energy metabolism, which led to a more severe energy deficit when receiving postnatal nutritional restriction. Moreover, the result revealed the negative impact of postnatal nutritional restriction in delaying myofiber maturation of the piglets through a reduction of the glycolytic fiber and an increase of the oxidative fiber, which could be associated with changing the metabolic status. Further research is needed to establish whether early nutrition intervention has long term effects in adult life.

## Figures and Tables

**Table 1:** Primer sequences of target and reference genes.

Gene	Primer Sequence (5′-3′)	Product (bp)	Genbank ID
*ACBP*	F: ATTCCAGGCATCCCACTTGG	92	NM_214119.1
	R: GCCACTACAAACAAGCGACC		
*CAT1*	F: TTGTCCAGGCAGAAGTAGGG	176	NM_001012613.1
	R: ACCTGCTTCTATGCCTTCGT		
*CAT2a*	F: GACCTGGGACTTGCTTTTGG	160	NM_001110420.1
	R: GATGTCCATTGGCACCCTTC		
*EAAT1*	F: TGATCCTCTTGTCCACCTGG	211	AY195622.1
	R: TACATGGCCACTGTCCTGAG		
*FABP3*	F: CTGCCATGGGTGAGTGTCAG	97	NM_001099931.1
	R: GCAAACTTGTCCACCTGCAG		
*FAT*	F: GAATGGATCCGGATAGCCCC	133	DQ192230.1
	R: GGCAGCTGTACCCCATCTCT		
*FATP1*	F: GACACAGTCCTCCCAGAAGC	130	NM_001083931.1
	R: GATCTATGACTGCCTGCCCC		
*GLUT1*	F: GTCCAGCCCTACGGATTAGC	109	XM_005665507.1
	R: CTTTACCCACATCCCACGCT		
*GLUT4*	F: GAATGCCAATGACGATGGCC	113	NM_001128433.1
	R: CTACTCAGGGCTGGCATCTG		
*LACD*	F: CCCATTCTGCGTGATGAGGA	149	D89478.1
	R: TGGAGGGGAGGAACGTCTAG		
*LACS*	F: TTCCTTCTGTTGGCTCGTCC	108	NM_001167629.2
	R: TCTCATGGACTCCTACGGCA		
*LAT1*	F: GGTCTGCAAAAGTCACAGCA	138	EF127856.1
	R: GGAATCTGCCCCTCTCCATT		
*POLG*	F: CTTTGAGGTTTTCCAGCAGCAG	119	XM_001927064.5
	R: GCTCCCAGTTTTGGTTGACAG		
*SSBP1*	F: CTTTGAGGTAGTGCTGTGTCG	143	XM_021078600.1
	R: CTCACCCCTGACGATGAAGAC		
*MyHC I*	F: AAGGGCTTGAACGAGGAGTAGA	130	AB053226
	R: TTATTCTGCTTCCTCCAAAGGG		
*MyHC IIa*	F: GCTGAGCGAGCTGAAATCC	155	AB025260
	R: ACTGAGACACCAGAGCTTCT		
*MyHC IIb*	F: ATGAAGAGGAACCACATTA	137	AB025261
	R: TTATTGCCTCAGTAGCTTG		
*MyHC IIx*	F: AGAAGATCAACTGAGTGAACT	113	AB025262
	R: AGAGCTGAGAAACTAACGTG		
*18S rRNA*	F: GACTCAACACGGGAAACCTCAC	146	AY265350.1
	R: ATCGCTCCACCAACTAAGAACG		
*β-Actin*	F: GGCGCCCAGCACGAT	66	DQ845171.1
	R: CCGATCCACACGGAGTACTTG		
*GAPDH*	F: TCGGAGTGAACGGATTTGGC	147	NM_001206359.1
	R: TGCCGTGGGTGGAATCATAC		

*ACBP*, acyl-CoA binding protein; *CAT*, cationic amino acid transporter; *EAAT1*, excitatory amino acid transporter 1; *FATP1*, fatty acid transport protein 1; *FAT*, fatty acid translocase; *FABP*, fatty acid binding protein; *GLUT*, glucose transporter; *LACS*, long-chain acyl-CoA synthetase; *LACD*, long-chain acyl-CoA dehydrogenase; *SSBP1*, single-stranded DNA-binding protein 1; *POLG*, DNA polymerase gamma; *LAT1*, L-type amino acid transporter 1; *MyHC*, myosin heavy chain; *GAPDH*, glyceral-dehyde-3-phosphate dehydrogenase.

**Table 2:** Effects of the level of nutrient intake on muscle weight of intrauterine growth-retarded (IUGR) and normal-birth weight (NBW) piglets.

Items	BW		NI		SEM	*p*-Value		
	IUGR	NBW	ANI	RNI		BW	NI	BW × NI
PM weight, g	13.5	22.6	19.8	16.3	2.2	<0.001	0.001	0.84
ST weight, g	13.5	25.7	21.1	18.2	2.4	<0.001	0.005	0.21
PM length, cm	8.6	10.3	10.1	8.7	0.8	0.001	<0.001	0.98
ST length, cm	6.0	7.3	6.9	6.4	0.7	0.006	0.05	0.36
Brain: PM	3.87	2.60	2.93	3.54	0.47	<0.001	0.004	0.24
Brain: ST	3.91	2.28	2.92	3.27	0.58	<0.001	0.15	0.91
PM: BW, %	0.27	0.30	0.29	0.28	0.03	0.007	0.16	0.12
ST: BW, %	0.27	0.34	0.31	0.31	0.03	<0.001	0.88	0.96

ANI, adequate nutrient intake; RNI, restricted nutrient intake; BW, birth weight; NI, nutrient intake. PM, psoas major muscle; ST, semitendinosus muscle.

**Table 3:** Effects of the level of nutrient intake on serum metabolite concentrations of intrauterine growth-retarded (IUGR) and normal-birth weight (NBW) piglets.

Items	BW		NI		SEM	*p*-Value		
	IUGR	NBW	ANI	RNI		BW	NI	BW × NI
tAA, μmol/L	95.55	84.10	92.85	86.80	9.96	0.03	0.24	0.40
Glucose, mmol/L	7.88	8.10	8.57	7.41	1.31	0.67	0.06	0.41
NEFA^1^, μmol/L	170.85	145.35	167.70	148.50	28.9	0.09	0.20	0.22
TG, mmol/L	0.90	0.95	1.06	0.79	0.25	0.71	0.05	0.89
TC, mmol/L	2.01	2.41	2.41	2.01	0.43	0.05	0.05	0.17
SUN, mmol/L	2.18	2.74	2.06	2.86	0.83	0.23	0.09	0.33
HDL-C, mmol/L	0.95	1.12	1.09	0.98	0.22	0.08	0.23	0.31
LDL-C, mmol/L	0.97	1.20	1.19	0.98	0.29	0.10	0.12	0.15
Leptin, ng/mL	1.68	1.30	1.45	1.53	0.13	<0.001	0.53	0.48
Insulin, μIU/mL	7.13	7.09	6.96	7.26	0.90	0.94	0.51	0.02
Glucose: insulin	1.13	1.15	1.25	1.03	0.08	0.63	0.14	0.05
T3, ng/mL	2.19	2.26	2.36	2.09	0.32	0.62	0.06	0.74
T4, ng/mL	42.65	38.89	39.20	42.34	5.23	0.11	0.17	0.42
T3/T4 (×10^−2^)	5.16	5.68	5.82	5.02	1.08	0.15	0.03	0.31

ANI, adequate nutrient intake; RNI, restricted nutrient intake; tAA, total amino acid; NEFA, non-esterified fatty acid; TG, triglyceride; TC, total cholesterol; HDL-C, high-density lipoprotein-cholesterol; LDL-C, low-density lipoprotein-cholesterol; T3, triiodothyronine; T4, tetraiodothyronine; BW, birth weight; NI, nutrient intake. ^1^ Data was transformed before statistical analysis.

**Table 4:** Effects of the level of nutrient intake on muscle metabolite content of psoas major muscles in intrauterine growth-retarded (IUGR) and normal-birth weight (NBW) piglets.

Items	BW		NI		SEM	*p*-Value		
	IUGR	NBW	ANI	RNI		BW	NI	BW × NI
Lactate, mmol/g prot	14.05	15.30	14.30	15.05	3.1	0.43	0.61	0.94
MDA, nmol/mg prot	1.97	1.88	1.96	1.88	0.18	0.36	0.39	0.24
TG, mmol/g prot	5.86	5.40	5.94	5.32	0.63	0.15	0.06	0.14
Glycogen, mg/g prot	4.75	5.22	5.49	4.48	0.38	0.02	<0.001	0.003
ATP, μmol/g prot	1870	2044	1962	1952	131	0.02	0.89	0.99

ANI, adequate nutrient intake; RNI, restricted nutrient intake; MDA, malondialdehyde; TG, triglyceride; prot, protein; ATP, adenosine triphosphate; BW, birth weight; NI, nutrient intake.

**Table 5:** Effects of the level of nutrient intake on myofiber characteristics of psoas major muscles in intrauterine growth-retarded (IUGR) and normal-birth weight (NBW) piglets.

Items	BW		NI		SEM	*p*-Value		
	IUGR	NBW	ANI	RNI		BW	NI	BW × NI
CSA, um^2^	139.3	234.2	221.0	152.5	26.6	<0.001	<0.001	<0.001
Myofiber diameter, um	24.9	25.2	25.3	24.7	2.2	0.83	0.48	0.03
Myofiber density, um^2^	2141	1889	1853	2177	379	0.16	0.08	0.65
Myofiber number, thousand	300.1	424.4	396.3	328.2	90.0	0.003	0.08	0.11

ANI, adequate nutrient intake; RNI, restricted nutrient intake; BW, birth weight; NI, nutrient intake; CSA, cross-sectional area.

**Table 6:** Effects of the level of nutrient intake on the contents of RNA, DNA, and protein in psoas major muscles of intrauterine growth-retarded (IUGR) and normal-birth weight (NBW) piglets.

Items	BW		NI		SEM	*p*-Value		
	IUGR	NBW	ANI	RNI		BW	NI	BW × NI
RNA, mg/g	1.30	1.39	1.66	1.03	0.18	0.26	<0.001	0.10
DNA, mg/g	1.21	1.20	1.23	1.18	0.06	0.90	0.10	0.53
Protein, mg/g	197.0	200.3	199.0	198.2	8.5	0.36	0.82	0.98
RNA:DNA	1.08	1.15	1.36	0.88	0.15	0.30	<0.001	0.06
Protein: DNA	156.6	155.7	152.1	160.2	17.3	0.53	0.24	0.63

ANI, adequate nutrient intake; RNI, restricted nutrient intake; BW, birth weight; NI, nutrient intake.

**Table 7:** Effects of the level of nutrient intake on metabolic enzyme activities in psoas major muscles of intrauterine growth-retarded (IUGR) and normal-birth weight (NBW) piglets.

Items	BW		NI		SEM	*p*-Value		
	IUGR	NBW	ANI	RNI		BW	NI	BW × NI
LDH, U/g protein	4997	5105	5069	5033	699	0.71	0.90	0.68
CS, U/g protein	1475	1564	1511	1529	132	0.12	0.75	0.32
HAD, U/g protein	761	745	703	803	72	0.60	0.003	0.57
LDH:CS	3.40	3.26	3.37	3.29	0.35	0.34	0.59	0.16
LDH:HAD	6.71	6.87	7.29	6.29	1.01	0.73	0.04	0.46
HAD:CS	0.52	0.48	0.47	0.53	0.05	0.07	0.02	0.76

ANI, adequate nutrient intake; RNI, restricted nutrient intake; BW, birth weight; NI, nutrient intake; LDH, lactate dehydrogenase; CS, citrate synthase; HAD, β-hydroxy-acyl-CoA-dehydrogenase.

**Table 8:** Effects of the level of nutrient intake on MyHC mRNA expressions and percentage in psoas major muscles of intrauterine growth-retarded (IUGR) and normal-birth weight (NBW) piglets.

Items	BW		NI		SEM	*p*-Value		
	IUGR	NBW	ANI	RNI		BW	NI	BW × NI
MyHC isoform mRNA expressions								
*MyHC I*	0.78	0.70	0.56	0.91	0.28	0.53	0.009	0.43
*MyHC IIa*	0.95	1.00	0.92	1.02	0.34	0.74	0.52	0.94
*MyHC IIb*	0.96	0.93	0.92	0.98	0.16	0.67	0.39	0.80
*MyHC IIx*	0.99	1.04	0.98	1.05	0.11	0.30	0.17	0.44
MyHC isoform percentages, %								
*MyHC I*	43.10	40.80	37.00	46.90	11.0	0.41	0.04	0.93
*MyHC IIa*	8.70	9.25	9.60	8.35	3.5	0.65	0.37	0.81
*MyHC IIb*	15.80	15.50	17.10	14.20	3.9	0.86	0.07	0.90
*MyHC IIx*	32.40	34.50	36.35	30.55	5.8	0.26	0.03	0.69

ANI, adequate nutrient intake; RNI, restricted nutrient intake; BW, birth weight; NI, nutrient intake. *MyHC*, myosin heavy chain.

## Supplementary Materials

The following are available online at https://www.mdpi.com/2076-2615/10/1/156/s1, Table S1: Composition and nutrient level of the basal formula milk powder (87.5% DM basis, %).
