# Supplementary material for: Effects of Birth Weight and Postnatal Nutritional Restriction on Skeletal Muscle Development, Myofiber Maturation, and Metabolic Status of Early-Weaned Piglets
Source: Animals (Basel). 2020 Jan 16;10(1):156. doi: 10.3390/ani10010156 (PMC7022288; doi:10.3390/ani10010156)
Supplement: Supplementary file 1 [file animals-10-00156-s001.pdf]

Supplementary table 1. Composition and nutrient level of the basal formula milk powder (87.5 % DM basis, %)

Ingredients	%
Whole-milk powder (24% CP)	58.00
Whey protein concentrate (34% CP)	25.00
Casein	5.70
Coconut oil	10.00
CaH2PO4	0.10
Choline chloride (50%)	0.10
Vitamin premix*	0.10
Mineral premix†	0.50
L-Arg (98.5%)	0.06
DL-Met (98.5%)	0.06
L-Lys·HCl (78.5%)	
L-Thr (98%)	0.03
L-Trp (98%)	0.05
Total	100.00
Nutrient content	
Digestible energy (kJ/kg)	18390
CP (%)	25.30
Ca (%)	1.02
Total P (%)	0.81
Available P (%)	0.67
Digestible Lys (%)	1.93
Digestible Met (%)	0.63
Digestible Arg (%)	0.86

CP, crude protein. * Vitamin premix provided per kg powder diet: vitamin A, 0.94 mg; vitamin D3, 0.01 mg; vitamin E, 20 mg; vitamin K3, 1 mg; vitamin B12, 0.04 mg; riboflavin, 5 mg; niacin, 20 mg; pantothenic acid, 15 mg; folic acid, 1.5 mg; thiamin, 1.5 mg; pyridoxine, 2 mg; biotin, 0.1 mg. † Mineral premix provided per kg powder diet: Zn, 90 mg; Mn, 4.0 mg; Fe, 90 mg; Cu, 6.0 mg; I, 0.2 mg; Se, 0.3 mg.
